# Supplementary material for: A Phase Ib Study of Durvalumab (MEDI4736) in Combination with Carbon-Ion Radiotherapy and Weekly Cisplatin for Patients with Locally Advanced Cervical Cancer (DECISION Study): The Early Safety and Efficacy Results
Source: Int J Mol Sci. 2023 Jun 23;24(13):10565. doi: 10.3390/ijms241310565 (PMC10342070; doi:10.3390/ijms241310565)
Supplement: Supplementary file 1 [file ijms-24-10565-s001.zip › ijms-2447321-supplementary.pdf]

Supplementary Table S1. Relevant concomitant care and interventions that are permitted or prohibited during the study

**Supportive medication/class of drug:**	**Usage:**
Concomitant medications or treatments (e.g., acetaminophen or diphenhydramine) deemed necessary to provide adequate prophylactic or supportive care, except for those medications identified as "prohibited," as listed above	To be administered as prescribed by the Investigator
Best supportive care (including antibiotics, nutritional support, correction of metabolic disorders, optimal symptom control, and pain management [including palliative radiotherapy to non-target lesions, etc.])	Should be used, when necessary, for all patients
Inactivated viruses, such as those in the influenza vaccine	Permitted
Laxatives and enemas	Permitted

**Prohibited medication/class of drug:**	**Usage:**
Any investigational anticancer therapy other than those under investigation in this study	Should not be given concomitantly whilst the patient is on study treatment and DLT evaluation period.
mAbs against CTLA-4, PD-1, or PD-L1 other than those under investigation in this study	Should not be given concomitantly whilst the patient is on study treatment and DLT evaluation period.
Any concurrent chemotherapy, radiotherapy, immunotherapy, or biologic or hormonal therapy for cancer treatment other than those under investigation in this study	Should not be given concomitantly whilst the patient is on study treatment and DLT evaluation period. (Concurrent use of hormones for non-cancer-related conditions [e.g., insulin for diabetes and hormone replacement therapy] is acceptable. Local treatment of isolated lesions, excluding target lesions, for palliative intent is acceptable [e.g., by local surgery or radiotherapy])

**Prohibited medication/class of drug:**	**Usage:**
Immunosuppressive medications including, but not limited to, systemic corticosteroids at doses exceeding <<10 mg/day>> of prednisone or equivalent, methotrexate, azathioprine, and tumor necrosis factor-α blockers	*Should not be given concomitantly, used for premedication prior to the I-O infusions or used in DLT evaluation period. The following are allowed exceptions:*<br>• *Use of immunosuppressive medications for the management of IP-related AEs,*<br>• *Short-term premedication for patients receiving combination agent durvalumab where the prescribing information for the agent requires the use of steroids for documented hypersensitivity reactions*<br>• *Use in patients with contrast allergies.*<br>• *In addition, use of inhaled, topical, and intranasal corticosteroids is permitted.*<br><br>*A temporary period of steroids will be allowed if clinically indicated and considered to be essential for the management of* non-immunotherapy related events experienced by *the patient (e.g., chronic obstructive pulmonary disease, radiation, nausea, etc.).*
EGFR TKIs	Should not be given concomitantly*.<br>Should be used with caution in the 90 days post last dose of durvalumab.<br>Increased incidences of pneumonitis (with third generation EGFR TKIs) and increased incidence of transaminase increases (with 1st generation EGFR TKIs) has been reported when durvalumab has been given concomitantly.
Live attenuated vaccines	Should not be given through 30 days after the last dose of IP (including SoC)
Herbal and natural remedies which may have immune-modulating effects	Should not be given concomitantly whilst the patient is on study treatment and DLT evaluation period.

**Prohibited medication/class of drug:**	**Usage:**
Blood transfusion (red blood cell concentrates, platelets), Granulocyte colony-stimulating factor (G-CSF)	Should not be given concomitantly whilst the patient is on study treatment and DLT evaluation period.<br>The following exceptions are allowed to avoid life threatening conditions (but it should be registered as DLT).<br>Hb < 6.5 g/dL<br>Platelet count < 25 × 10^9/L (<25,000 per mm3)

* The word "concomitantly" here means from the start date of the study treatment to within 4 weeks after the last administration of durvalumab.

Supplementary Table S2. Inclusion and exclusion criteria of the present study

Inclusion Criteria: Patients must fulfill all of the following criteria

- Capable of giving signed informed consent which includes compliance with the requirements and restrictions listed in the informed consent form (ICF) and in this protocol. Written informed consent and any locally required authorization obtained from the patient/legal representative prior to performing any protocol-related procedures, including screening evaluations.
- Age ≥20 years for Japanese patients.
- Age ≤ 75 years at time of study entry.
- Histologically proven uterine cervical cancer; Stage IIB, IIIA, IIIB, IIIC1, and IVA in FIGO (2018) staging.
- Eastern Cooperative Oncology Group (ECOG) performance status of 0 or 1
- Body weight >30 kg
- Adequate normal organ and marrow function as defined below:
- Haemoglobin ≥9.0 g/dL
- Absolute neutrophil count (ANC) ≥1000 per mm3
- Platelet count ≥75 × 109/L (≥75,000 per mm3)
- Serum bilirubin ≤1.5 x institutional upper limit of normal (ULN). <<This will not apply to patients with confirmed Gilbert's syndrome (persistent or recurrent hyperbilirubinemia that is predominantly unconjugated in the absence of hemolysis or hepatic pathology), who will be allowed only in consultation with their physician.>>
- AST (SGOT)/ALT (SGPT) ≤2.5 x institutional upper limit of normal unless liver metastases are present, in which case it must be ≤5x ULN
- Measured creatinine clearance (CL) ≥40 mL/min or Calculated creatinine CL≥40 mL/min by the Cockcroft-Gault formula (Cockcroft and Gault 1976) or by 24-hour urine collection for determination of creatinine clearance:

Females:

$$\text{Creatinine CL (mL/min)} = \frac{\text{Weight (kg) x (140 – Age)}}{\text{72 x serum creatinine (mg/dL)}} \times 0.85$$

- Patient is willing and able to comply with the protocol for the duration of the study including undergoing treatment and scheduled visits and examinations including follow up.
- Must have a life expectancy of at least 12 weeks
- No prior chemotherapy or radiotherapy for cervical cancer.
- Tumor assessment by computed tomography (CT) scan or magnetic resonance imaging (MRI) must be performed within 28 days prior to.

Exclusion Criteria: Patients should not participate if any of the following exclusion criteria are fulfilled

- Participation in another clinical study with an investigational product during the last 3 months.
- Concurrent enrolment in another clinical study, unless it is an observational (non-interventional) clinical study or during the follow-up period of an interventional study
- Receipt of the last dose of anticancer therapy (chemotherapy, immunotherapy, endocrine therapy, targeted therapy, biologic therapy, tumor embolization, monoclonal antibodies) ≤1 years prior to the first dose of study drug.
- Any unresolved toxicity NCI CTCAE Grade ≥2* from previous anticancer therapy with the exception of alopecia, vitiligo, and the laboratory values defined in the inclusion criteria

  *Patients with Grade ≥2 neuropathy will be evaluated on a case-by-case basis after consultation with the Study Physician. Patients with irreversible toxicity not reasonably expected to be exacerbated by treatment with durvalumab may be included only after consultation with the Study Physician.
- Any concurrent chemotherapy, IP, biologic, or hormonal therapy for cancer treatment.

Concurrent use of hormonal therapy for non-cancer-related conditions (e.g., hormone replacement therapy) is acceptable.

- Radiotherapy treatment to more than 30% of the bone marrow or with a wide field of radiation within 4 weeks of the first dose of study drug.
- Major surgical procedure (as defined by the Investigator) within 28 days prior to the first dose of IP. Note: Local surgery of isolated lesions for palliative intent is acceptable.
- History of allogenic organ transplantation.
- Active or prior documented autoimmune or inflammatory disorders (including inflammatory bowel disease [e.g., colitis or Crohn's disease], diverticulitis [with the exception of diverticulosis], systemic lupus erythematosus, Sarcoidosis syndrome, or Wegener syndrome [granulomatosis with polyangiitis, Graves' disease, rheumatoid arthritis, hypophysitis, uveitis, etc]). The following are exceptions to this criterion:
    - ✓ Patients with vitiligo or alopecia
    - ✓ Patients with hypothyroidism (e.g., following Hashimoto syndrome) stable on hormone replacement
    - ✓ Any chronic skin condition that does not require systemic therapy
    - ✓ Patients without active disease in the last 5 years may be included but only after consultation with the study physician
    - ✓ Patients with celiac disease controlled by diet alone
- Uncontrolled intercurrent illness, including but not limited to, ongoing or active infection, symptomatic congestive heart failure, uncontrolled hypertension, unstable angina pectoris, cardiac arrhythmia, interstitial lung disease, serious chronic gastrointestinal conditions associated with diarrhea, or psychiatric illness/social situations that would limit compliance with study requirement, substantially increase risk of incurring AEs or compromise the ability of the patient to give written informed consent
- History of another primary malignancy except for
    - ✓ Malignancy treated with curative intent and with no known active disease ≥5 years before the first dose of IP and of low potential risk for recurrence.
    - ✓ Adequately treated non-melanoma skin cancer or lentigo maligna without evidence of disease.
    - ✓ Adequately treated carcinoma in situ without evidence of disease.
- History of leptomeningeal carcinomatosis
- History of active primary immunodeficiency
- Active infection including **tuberculosis** (clinical evaluation that includes clinical history, physical examination and radiographic findings, and TB testing in line with local practice), **hepatitis B** (known positive HBV surface antigen (HBsAg) result), **hepatitis C.** Patients with a past or resolved HBV infection (defined as the presence of hepatitis B core antibody [anti-HBc] and absence of HBsAg) are eligible. Patients positive for hepatitis C (HCV) antibody are eligible only if polymerase chain reaction is negative for HCV RNA.
- Current or prior use of immunosuppressive medication within 14 days before the first dose of durvalumab. The following are exceptions to this criterion:
    - ✓ Intranasal, inhaled, topical steroids, or local steroid injections (e.g., intra articular injection)
    - ✓ Systemic corticosteroids at physiologic doses not to exceed <<10 mg/day>> of prednisone or its equivalent
    - ✓ Steroids as premedication for hypersensitivity reactions (e.g., CT scan premedication)
- Receipt of live attenuated vaccine within 30 days prior to the first dose of IP. Note: Patients, if enrolled, should not receive live vaccine whilst receiving IP and up to 30 days after the last dose of IP.
- Female patients who are pregnant or breastfeeding or female patients of reproductive potential who are not willing to employ effective birth control from screening to 90 days after the last dose of durvalumab monotherapy.
- Known allergy or hypersensitivity to any of the study drugs or any of the study drug excipients.

- Prior randomisation or treatment in a previous durvalumab clinical study regardless of treatment arm assignment.
- Patients who have received prior anti–PD-1, anti PD-L1 or anti CTLA-4.
- Resectable uterine cervical cancer.
- Recurrence of uterine cervical cancer.
- Patients with intestinal invasion of cervical cancer.
- Uncontrollable pain due to cervical cancer.
- Patients who have active or history of severe interstitial pneumonia or pulmonary fibrosis.
- Patients with ileus.
- Patients with systemic infection which required intensive treatment.
- Patients with a history of transient ischemic stroke, cerebrovascular accident, thrombosis, or thromboembolism within 180 days before the enrolment of this study.
- Patients with uncontrolled diabetes or bleeding tendency.
- Judgment by the investigator that the patient is unsuitable to participate in the study and the patient is unlikely to comply with study procedures, restrictions and requirements.
